# Superior Enhancement of Cutaneous Microcirculation Due to “Cyclic” Application of a Negative Pressure Wound Therapy Device in Humans – Local and Remote Effects

**DOI:** 10.3389/fsurg.2022.822122

**Published:** 2022-03-03

**Authors:** Alexander Sogorski, Amira Becker, Mehran Dadras, Christoph Wallner, Johannes Maximillian Wagner, Maxi v Glinski, Marcus Lehnhardt, Björn Behr

**Affiliations:** Department of Plastic Surgery and Hand Surgery, Burn Center, BG University Hospital Bergmannsheil Bochum, Ruhr-University Bochum, Bochum, Germany

**Keywords:** Negative Pressure Wound Therapy (NPWT), cutaneous microcirculation, wound healing, remote conditioning, Vacuum-assisted closure (VAC)

## Abstract

**Objectives:**

Despite a common utilization of “Negative Pressure Wound Therapy” (NPWT) Devices in a wide range of specialties, some of the basic mechanisms of action of the techniques are still on debate. Conflicting results from prior studies demonstrate our lack of understanding how wound-bed perfusion or cutaneous microcirculation is affected by NPWT.

**Methods:**

We conducted a prospective randomized study which included 45 healthy subjects to further investigate the acute effects of NPWT on cutaneous microcirculation underneath the applied dressing. Three modes of application, namely, continuous, intermittent, cyclic, were tested. Amongst others, measurements of elicited surface pressure and a comprehensive microcirculatory analysis were carried out by utilizing an O2C-device. For the detection of (systemic) remote effects, perfusion changes of the contra-lateral thigh were evaluated.

**Results:**

All three tested modes of application led to a significant (*p* < 0.05) improvement in local tissue perfusion with an increased blood flow of max +151% and tissue oxygen saturation of +28.2% compared to baseline values. Surface pressure under the dressing significantly increased up to 29.29 mmHg due to the activation of the NPWT device. Continuous, intermittent, and cyclic application of negative pressure were accurately sensed by participants, resulting in reported pain values that mirrored the different levels of applied suction. Although the cyclic application mode showed the most pronounced effects regarding microcirculatory changes, no statistical significance between groups was observed.

**Conclusion:**

We could demonstrate a significant improvement of cutaneous microcirculation under an applied NPWT dressing with favorable effects due to cyclic mode of application. An increased surface pressure leads to a better venous drainage of the tissue, which was shown to increase arterial inflow with a consecutive improvement of oxygen supply. Further research is warranted to evaluate our findings regarding wound bed perfusion in the clinical field with respect to formation of granulation tissue and wound healing.

## Introduction

Over the past decades, the application of “negative pressure” has evolved to a cornerstone in the treatment of acute and chronic wounds in almost all specialties. Various available synonyms reflect the past developments and current applications of the technique involving, amongst others, “Vacuum-assisted closure” (VAC), “Negative Pressure Wound Therapy” (NPWT), “closed incision Negative Pressure Therapy” (ciNPT), or “Negative Pressure Wound Therapy with instillation” (NPWTi) (1, 2). All but ciNPT are used for treatment of open wounds and exert the known beneficial effects of “negative pressure” therapy on wound healing, i.e., sufficient temporary wound closure, promotion of wound bed granulation, mechanical contraction and stabilization of wound margins, and efficient reduction of bacterial load.

Wound bed perfusion represents another key factor in wound healing. Effects of “negative pressure” on wound bed perfusion have lately been widely discussed. Results from different research groups have partly shown diverging results which could seriously question the hypothesis of an enhancement of local and adjacent wound bed perfusion due to application of a negative pressure dressing (2–5). Actual doubt was risen based on the physically driven understanding of a compression of underlying tissues through application of a negative pressure dressing, particularly, on the capillary network that is subjected to surface pressure. Consecutively, occlusion of microvessels would result in a diminished rather than enhanced capillary blood flow, causing local hypoxia and, probably, ischemia. Moreover, the utilization of an otherwise broadly used technique for perfusion analysis, laser-doppler velocimetry, was questioned to be flawed due to the impact of “pressure-artifacts” (6), therefore resulting in a false-positive sign of an enhancement in perfusion underneath an applied NPWT dressing.

On the contrary, current research regarding perfusion alterations due to ciNPT and the application of negative pressure wound therapy over closed incisions found that blood flow and consecutive tissue oxygenation acutely improved upon treatment (7–9). Additionally, NPWT was also successfully applied in free tissue transfer, with a reduction of postoperative tissue damage instead of an increment (10). No adverse effects of negative pressure were found. In a previous analysis, we used continuous laser-doppler flowmetry combined with white-light spectroscopy for a comprehensive real-time analysis of microcirculatory changes under an NPWT dressing (8). Application of an intermittent negative pressure resulted in a stepwise increase in local tissue perfusion with a consecutive enhancement of tissue oxygen saturation.

We set up this prospective randomized comparative study to investigate the acute effects of different levels of applied suction on the cutaneous microcirculation in humans. Furthermore, we wanted to test the hypothesis of superior effects due to different pressure distribution modalities (continuous, intermittent, and cyclic) during NPWT. Beyond acute local effects, we also targeted possible “remote conditioning” effects with respect to potential overlaps in triggering of “global” effects as observed in the emerging concept of Remote Ischemic Conditioning (RIC).

This preclinical study is part of a two-stage approach to investigate the link between acute alterations of wound bed perfusion and actual wound healing in patients during NPWT.

## Materials and Methods

The presented study was approved by the local ethics committee of the Ruhr-University Bochum (Reg. number 18-6689, 18.10.2019) and carried out in accordance with the Declaration of Helsinki. All actions were conducted at the BG University Hospital Bergmannsheil Bochum, Germany. All measurements were conducted by the same investigator.

Forty-five young healthy volunteers without any medical history of chronic diseases or active medication, except for oral contraceptives, were included into the study. All participants were non-smokers. Written informed consent was obtained and general characteristics were recorded due to anamnesis.

Vital parameters were measured after the intervention to avoid any interference with the NPWT stimulus.

All subjects were randomly assigned to one of the three 60-min test groups with a different amount/duration of applied suction (1: −120 mmHg for 60 min, 2: 3x −120/0 mmHg for 10/10 min, 3: 3x −120/−60 mmHg for 10/10 min). Participants were comfortably placed in a supine position in a temperature-controlled room at 22°C.

Measurements of cutaneous microcirculation were carried out on each anterior lateral thigh in every participant. One probe of an O2C-device (“Oxygen-to-see” ©LEA Medizintechnik Giessen, Germany) was attached to the intact skin with double-sided adhesive tapes, as provided by the manufacturer, centrally under the foam, and another was placed on the contralateral thigh. Penetration depth of both probes was 1–2 mm. Both probes were placed central to an imaginary line between the anterior superior iliac spine and the lateral margin of the patella.

In brief, the O2C-device utilizes combined laser-doppler and white-light spectroscopy for a comprehensive real-time analysis of local microcirculation. Given a penetration depth of 1–2 mm, cutaneous capillary network can be investigated with regards to blood flow (BF), velocity (VELO), postcapillary oxygen saturation (StO2), and relative hemoglobin content (rHb).

**Figure 1:**
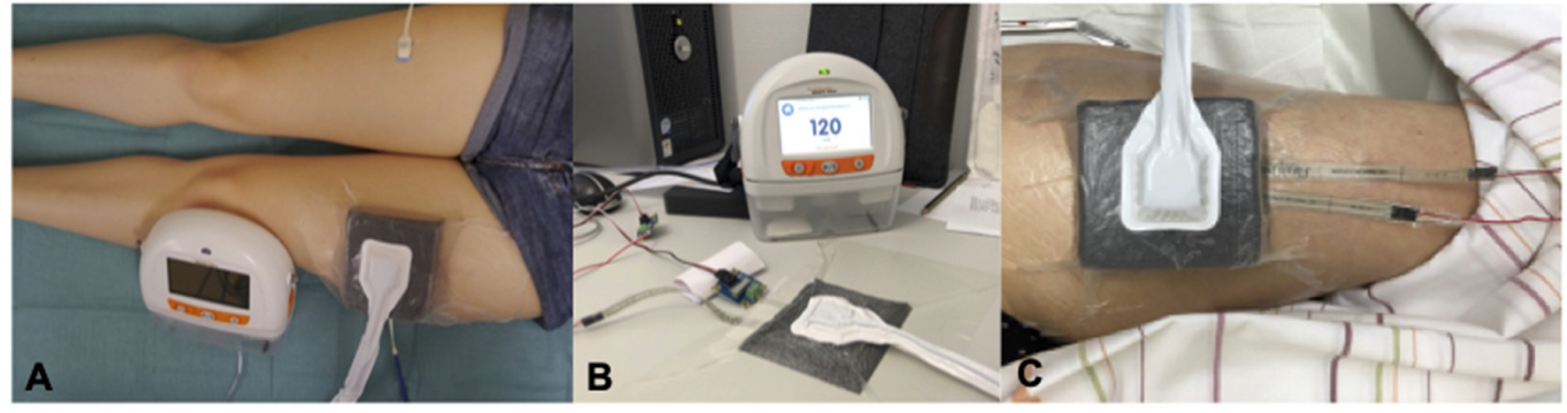
Study Set-up. **(A)** Microcirculatory Analysis via two O2C-probes under an applied NPWT-dressing and on the contralateral thigh. **(B)** Calibration-process of pressure probes under controlled conditions on a firm surface. **(C)** Analysis of surface pressure under an applied NPWT-dressing via two probes located central/marginal.

We used a RENASYStouch system (©Smith and Nephew Medical Ltd. GB) to investigate the effects of different applications of “negative pressure” (“suction”) on cutaneous microcirculation on intact skin. A RENASYS-F Polyurethane Foam (©Smith and Nephew Medical Ltd. GB) was cut to 10 x 10 cm strips, placed on one thigh for central coverage of the O2C probe, and attached to the adjacent skin via adhesive foil. Connection between the dressing and the NPWT device was carried out according to instructions for use provided by the manufacturer (Figure 1).

After completion of the study set-up, participants rested for 5 min before baseline measurements of cutaneous microcirculation were started. After 10 min, the NPWT device was activated according to prior randomization for a total of 60 min. After completion of the suction-period, the NPWT device was disconnected with the foam still in place, and measurements of cutaneous microcirculation were continued for another 30 min.

Discomfort or pain due to the NPWT dressing was frequently assessed, utilizing a numeric pain scale (0 = no pain – 10 = maximum imaginable pain).

In a separate series of measurements, a FlexiForce (©Tekscan, Inc South Boston, United States) force sensor (0-1lb) was placed under the foam instead of the O2C probe, and repeated measurements of different amounts of suction were carried out. Data from the sensor (change of resistance) was transformed to mmHg after calibration-measurements by means of additional laboratory testing.

### Statistical Analysis

Statistical analysis was carried out using the Microsoft Excel and the IBM SPSS 26 commercially available software. All descriptive parameters and pain-values are presented as mean values (± SD), and the Kruskal-Wallis test was used for comparison of groups. A *p* < 0.05 was considered significant.

Changes of microcirculatory parameters due to NPWT were calculated as relative changes compared to baseline values. Statistical significance was determined by the confidence interval method. Data is shown as mean and 95% confidence interval. For correction of short-term artifacts of continuous microcirculation measurements (one data point per second), these data were separated into corresponding intervals of 5 min length, and linear regression analysis was performed to create a corrected slope. Testing for normal distribution via the Shapiro-Wilk test showed non-uniform results; hence, the non-parametric Kruskal-Wallis test was used for comparison between groups. A *p* < 0.05 was considered significant. Lastly, the Dunn-Bonferroni test was used for *post-hoc* analysis.

The difference between surface pressure was calculated using ANOVA (repeated testing) and the Greenhouse-Geisser-correction.

## Results

### General Characteristics

Twenty-five female and 20 male subjects were included in the study. The mean age of all participants was 23.98 ± 2.78 years (range: 20–32), and mean BMI was 22.92 ± 4.1 kg/m^2^ (range: 16.6–42.9). Assessed vital parameters showed physiologic values and randomization comparison between groups showed no relevant differences (*p* > 0.05). See Table 1 for complete data.

**Table 1:** General characteristics.

	**All**	**Group 1 cont.**	**Group 2 intermitt**.	**Group 3 cycl.**	***p***
Age (Y)	23.98 ± 2.78	24.47 ± 3.22	24.53 ± 3.14	22.93 ± 1.53	0.42
Sex (f /m)	25/20	8/7	8/7	9/6	0.916
BMI (kg/m^2^)	22.92 ± 4.1	24.48 ± 5.99	21.7 ± 2.95	22.57 ± 1.97	0.312
Hight (cm)	174.49 ± 9.56	172.33 ± 9.96	175.00 ± 9.59	176.13 ± 9.39	0.474
Weight (kg)	70.07 ± 14.64	73.07 ± 19.60	66.87 ± 12.97	70.27 ± 10.04	0.775
BP sys	116.07 ± 12.62	120.00 ± 15.35	113.33 ± 10.12	114.87 ± 11.66	0.325
BP dia	79.56 ± 8.31	82.00 ± 10.49	78.00 ± 7.97	78.67 ± 5.82	0.465
HR (bpm)	66.71 ± 9.7	69.87 ± 11.03	66.00 ± 11.36	64.27 ± 5.23	0.341
SpO_2_ (%)	98.07 ± 0.39	98.33 ± 0.488	97.93 ± 0.26	97.93 ± 0.26	0.005
Temp (°C)	35.46 ± 0.58	35.15 ± 0.64	35.66 ± 0.52	35.53 ± 0.47	0.104

*General characteristics of all participants and separated according to randomization (BMI, body mass index, BP, Blood pressure, sys, systolic, dia, diastolic, HR, heartrate)*.

### Microcirculation

#### Blood Flow (BF)

**Figure 2:**
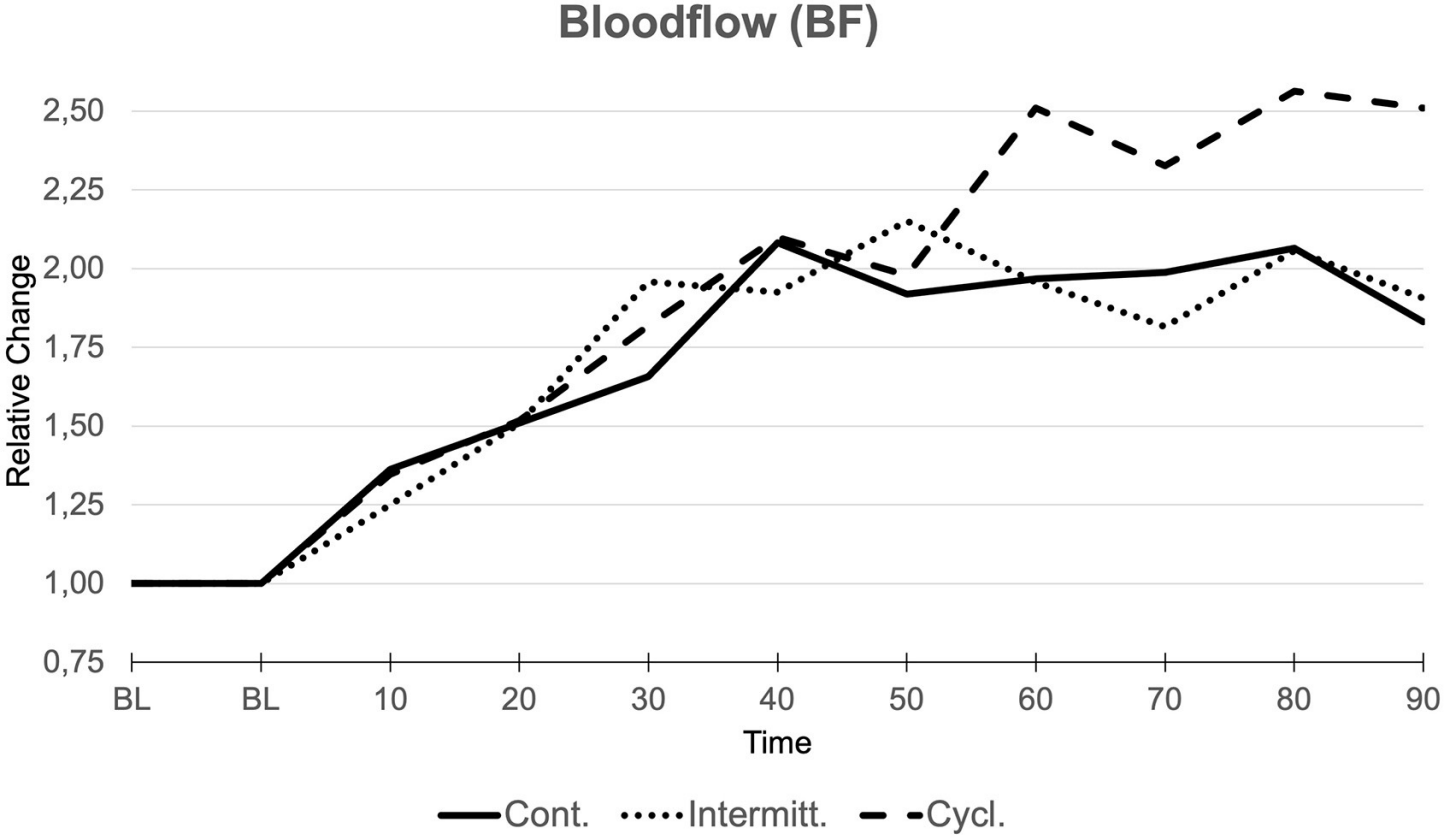
Bloodflow (BF). Relative changes compared to baseline values (BL, 1.000) over the course of measurements (min) in all three groups.

Regardless of the application of different pressure levels, intervals of suction and cutaneous blood flow below the foam dressing was significantly enhanced in all three groups (Figure 2). BF increased in a virtually linear trend during the first 60 min after activation of “negative pressure” in all groups, regardless of the chosen modality of up to +96.7% (CI: 1.543–2.391) for group 1, +95.6% (CI: 1.487–2.425) for group 2, and +151.0% (CI: 1.682–3.337) for group 3 compared to baseline values (BL). Peak values were reached after the NPWT device was disconnected in group 1 and 3 with a maximum increase of +108.7% (CI: 1.604–2.570) for group 1 (75–85 min after BL) and +156.4% (CI: 1.869–3.259) for group 3 (80–85 min after BL). In group 2, peak values were reached earlier, specifically when suction was finally turned off during the 3rd interval (45–55 min after BL) with a maximum increase of +115.3% (CI: 1.684–2.621).

Comparison of groups via the Kruskal-Wallis test showed no significant differences between groups.

#### Post-capillary Tissue Oxygen Saturation (StO_2_)

**Figure 3:**
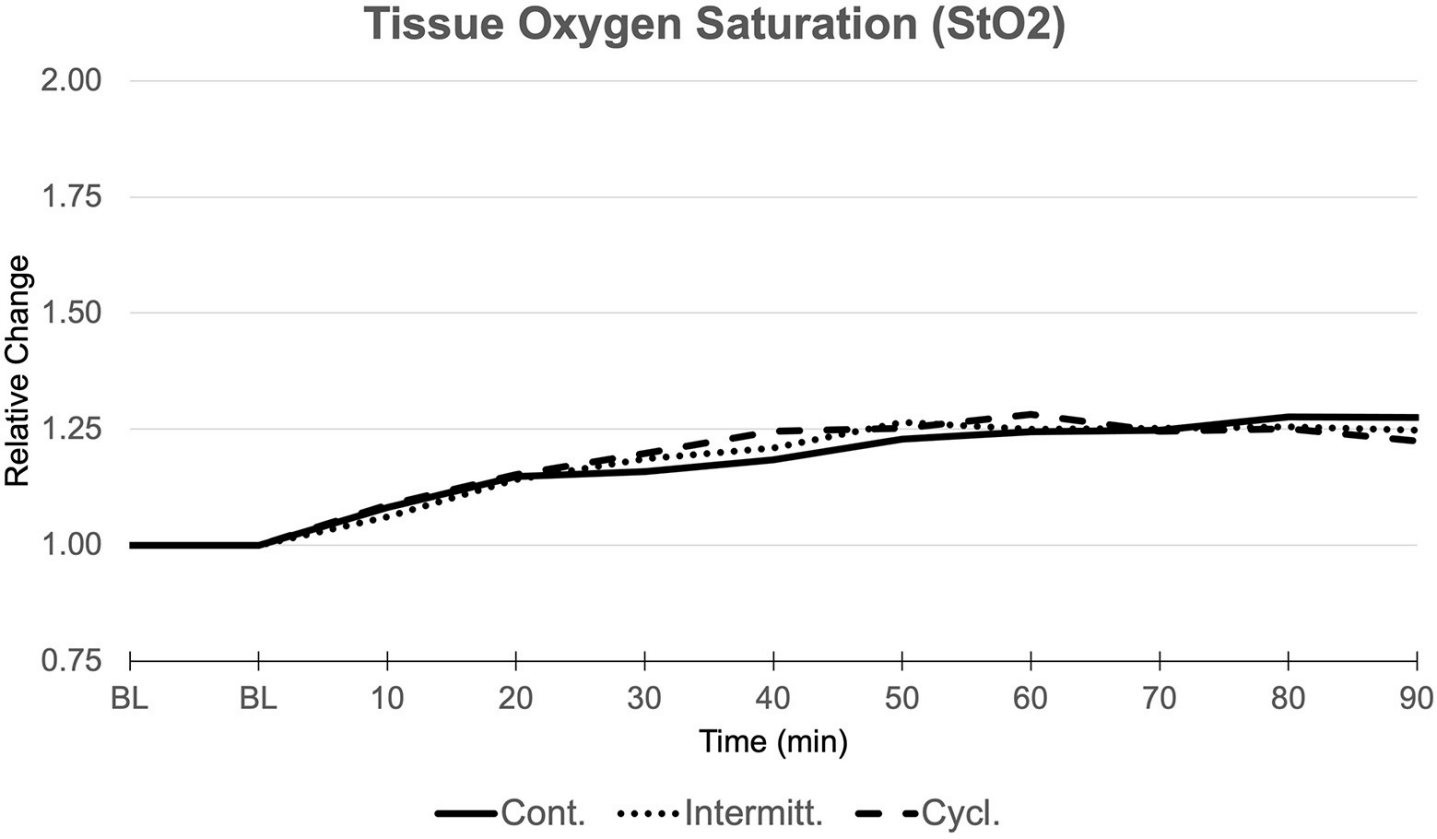
Tissue Oxygen Saturation (StO_2_). Relative changes compared to baseline values (BL, 1.000) over the course of measurements (min) in all three groups.

Corresponding to enhancements in cutaneous BF, StO_2_-values steadily increased when suction was active (Figure 3). Similar to BF values, peak levels were reached when suction was finally tuned off at the end of the suction period in all three groups. We observed a maximum significant increase in StO_2_ of +28.1% (CI: 1.162–1.400) for group 1, +26.4% (CI: 1.205–1.324) for group 2, and +28.2% (CI 1.167–1.397) for group 3 compared to BL.

Comparison between groups showed no significant differences.

#### Relative Hemoglobin Content (rHb) and Red Blood Cell Velocity (VELO)

Both parameters were significantly altered due to the NPWT stimulus. The rHb steadily increased to a maximum of +16.5% (CI: 1.125–1.205) for group 1, +16.3% (CI: 1.126–1.201) for group 2, and +14.7% (1.114–1.179) for group 3 compared to BL. The V was observed to increase up to a max of +18.9% (CI 1.069–1.310) during active suction in group 1 and was further enhanced after suction was finally turned off [+21.7 % (CI: 1.079–1.356)]. For group 2, peak values were observed after suction was turned off during the third period with a maximum increase in VELO of +21.0% (CI: 1.056–1.363). In group 3, the greatest change in VELO was found after completion of the active-suction period of around 75 min after BL with a maximum increase of +36.3% (CI: 1.207–1.518).

No significant differences in acute changes between groups were observed in rHb and V.

See Table 2 for complete microcirculatory data.

**Table 2:** Microcirculatory data (Negative Pressure Wound Therapy, NPWT).

	**Group 1 (continuous)**	**Group 2 (intermittent)**	**Group 3 (cyclic)**	***p*-value**
	**Δ % vs. baseline (CI)**	**Δ % vs. baseline (CI)**	**Δ % vs. baseline (CI)**	
**Blood flow (BF)**				
BL + 10 min	+36.3 (1.182–1.544)	+19.9 (1.034–1.364)	+34.7 (1.212–1.482)	0.580
+20 min	+51.0 (1.202–1.819)	+50.8 (1.327–1.690)	+51.9 (1.304–1.733)	0.998
+30 min	+65.7 (1.354–1.960)	+95.8 (1.613–2.303)	+81.9 (1.481–2.158)	0.482
+40 min	+108.2 (1.552–2.611)	+92.5 (1.566–2.285)	+109.9 (1.655–2.543)	0.840
+50 min	+91.9 (1.491–2.348)	+115.1 (1.660–2.641)	+116.3 (1.654–2.672)	0.726
+60 min	+96.7 (1.543–2.391)	+95.6 (1.487–2.425)	+110.1 (1.553–2.649)	0.901
+70 min	+98.8 (1.528–2.447)	+81.6 (1.338–2.295)	+132.7 (1.794–2.860)	0.357
+80 min	+106.4 (1.615–2.514)	+105.7 (1.462–2.652)	+156.4 (1.869–3.259)	0.499
+90 min	+83.1 (1.454–2.207)	+90.7 (1.395–2.418)	+151.0 (1.865–3.156)	0.165
**Post-capillary tissue oxygen saturation (StO**_**2**_ **)**				
BL + 10 min	+9.7 (1.066–1.129)	+7.3 (1.037–1.110)	+8.6 (1.051–1.121)	0.627
+20 min	+14.8 (1.107–1.190)	+14.2 (1.090–1.193)	+15.2 (1.092–1.213)	0.962
+30 min	+19.2 (1–147–1.237)	+18.6 (1.116–1.257)	+19.8 (1.124–1.271)	0.972
+40 min	+18.4 (1.105–1.263)	+21.0 (1.142–1.277)	+24.6 (1.163–1.329)	0.540
+50 min	+22.9 (1.125–1.335)	+26.4 (1.205–1.324)	+25.1 (1.181–1.322)	0.832
+60 min	+24.5 (1.140–1.350)	+24.9 (1.196–1.302)	+22.9 (1.151–1.308)	0.940
+70 min	+24.7 (1.150–1.345)	+25.2 (1.195–1.309)	+24.6 (1.176–1.315)	0.993
+80 min	+27.6 (1.169–1.383)	+25.5 (1.186–1.324)	+25.1 (1.168–1.333)	0.911
+90 min	+27.5 (1.157–1.392)	+24.8 (1.178–1.318)	+18.8 (1.136–1.239)	0.393
**Relative hemoglobin content (rHb)**				
BL + 10 min	+5.9 (1.037–1.080)	+5.7 (1.038–1.076)	+4.9 (1.031–1.067)	0.763
+20 min	+8.1 (1.065–1.098)	+8.2 (1.055–1.108)	+8.8 (1.062–1.114)	0.909
+30 min	+11.7 (1.091–1.143)	+13.1 (1.099–1.162)	+11.3 (1.078–1.148)	0.344
+40 min	+12.8 (1.102–1.154)	+13.6 (1.100–1.172)	+14.2 (1.105–1.179)	0.910
+50 min	+15.3 (1.116–1.189)	+16.3 (1.126–1.201)	+12.7 (1.099–1.155)	0.350
+60 min	+16.4 (1.125–1.203)	+15.4 (1.112–1.195)	+15.5 (1.115–1.195)	0.923
+70 min	+15.8 (1.122–1.195)	+14.7 (1.109–1.185)	+13.5 (1.104–1.165)	0.644
+80 min	+16.5 (1.125–1.205)	+14.3 (1.102–1.184)	+14.7 (1.114–1.179)	0.690
+90 min	+15.9 (1.133–1.185)	+14.0 (1.096–1.184)	+14.7 (1.113–1.181)	0.766
**Red blood cell velocity (VELO)**				
BL + 10 min	+4.8 (1.007–1.090)	+6.3 (0.990–1.137)	+9.3 (1.033–1.153)	0.599
+20 min	+5.2 (1.000–1.104)	+10.3 (1.038–1.168)	+13.7 (1.058–1.217)	0.245
+30 min	+5.8 (0.995–1.122)	+17.0 (1.080–1.259)	+22.6 (1.109–1.343)	0.069
+40 min	+18.9 (1.069–1.310)	+16.7 (1.058–1.277)	+25.4 (1.133–1.376)	0.576
+50 min	+14.1 (1.048–1.235)	+20.3 (1.077–1.329)	+22.8 (1.129–1.322)	0.516
+60 min	+16.3 (1.057–1.270)	+17.2 (1.045–1.300)	+21.4 (1.105–1.322)	0.818
+70 min	+14.1 (1.033–1.248)	+19.3 (1.027–1.359)	+35.8 (1.211–1.504)	0.089
+80 min	+21.7 (1.079–1.356)	+20.2 (1.044–1.360)	+35.6 (1.211–1.502)	0.213
+90 min	+21.6 (1.105–1.326)	+17.6 (1.041–1.311)	+35.8 (1.205–1.510)	0.152

*Relative changes compared to baseline values (BL) of all parameters, CI (Confidence interval) > 1.000 indicating significant change compared to BL, p-values for comparison between groups*.

## Pain/Discomfort

**Figure 4:**
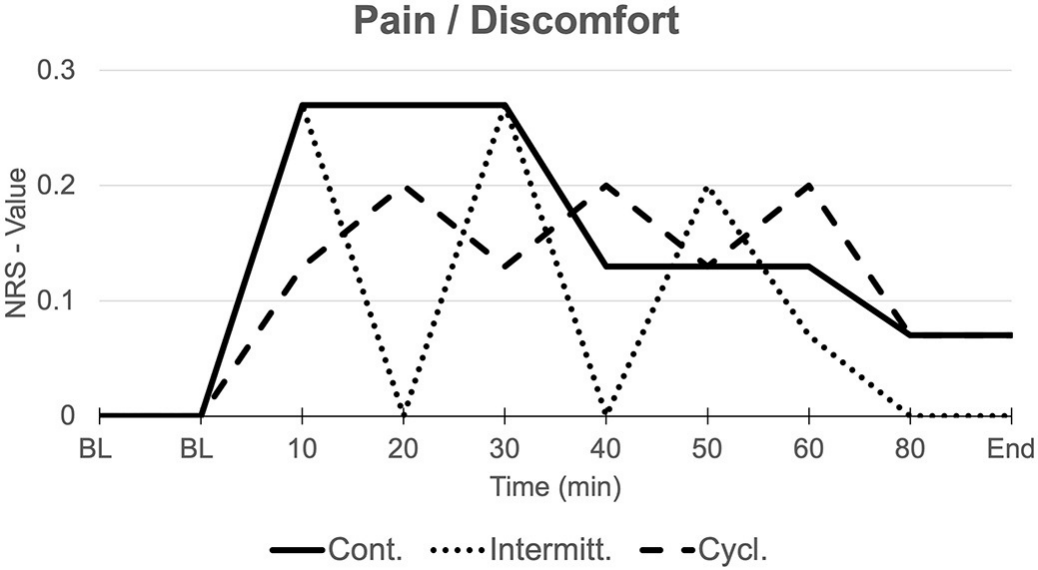
Discomfort/Pain. Mean elicited discomfort, respectively pain (NRS) in all three groups over the course of measurements. Mode of applied suction (continuous, intermittent, cyclic) is mimicked by participants sensation.

As expected, reported levels of discomfort were nominal. No statistic difference was found in comparison of maximum values between groups (*p* > 0.05). Beyond that, the mode of applied suction (continuous, intermittent, and cyclic) was mimicked by participants sensation and therefore could be related to apparent discomfort as demonstrated in Figure 4.

## Surface Pressure

**Figure 5:**
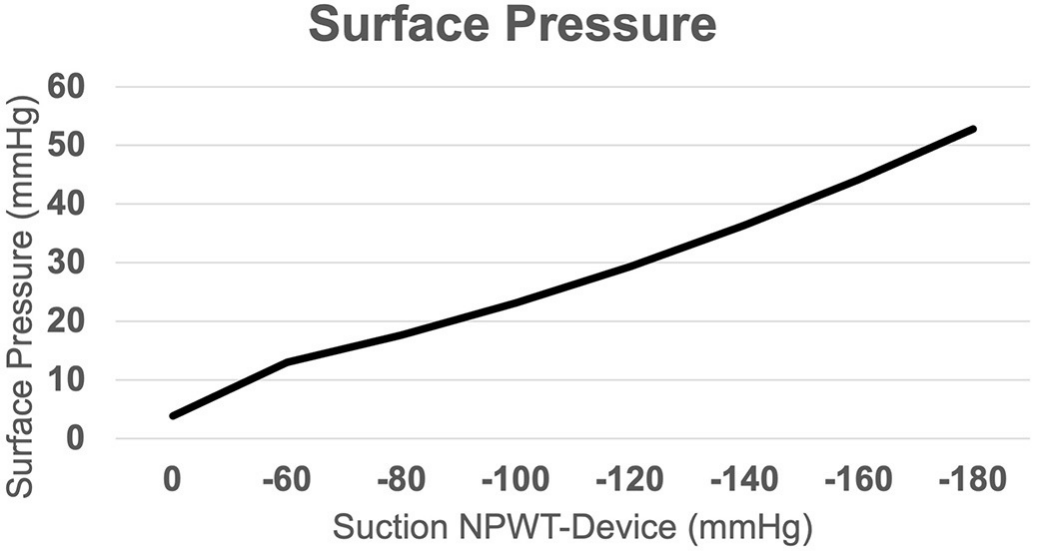
Surface Pressure. Resulting surface pressure due to applied suction via the NPWT-device, indicating an almost linear correlation between applied suction and positive pressure acting on the skin surface.

Applied suction caused significant changes in the surface pressure (sp) of the underlying skin. At baseline condition, when suction was off, a positive pressure of 3.872 mmHg due to the attached dressing could be observed. Activation of suction resulted in a gradual increase of compressional forces toward the skin with 12.980 mmHg at −60 mmHg and 29.288 mmHg at −120 mmHg. Comparison between these different stages showed a significant increase in surface pressure for 0 mmHg vs. −60 mmHg (*p* = 0.001) and −60 mmHg vs. −120 mmHg (*p* = 0.015; Figure 5).

## Remote Effects

**Figure 6:**
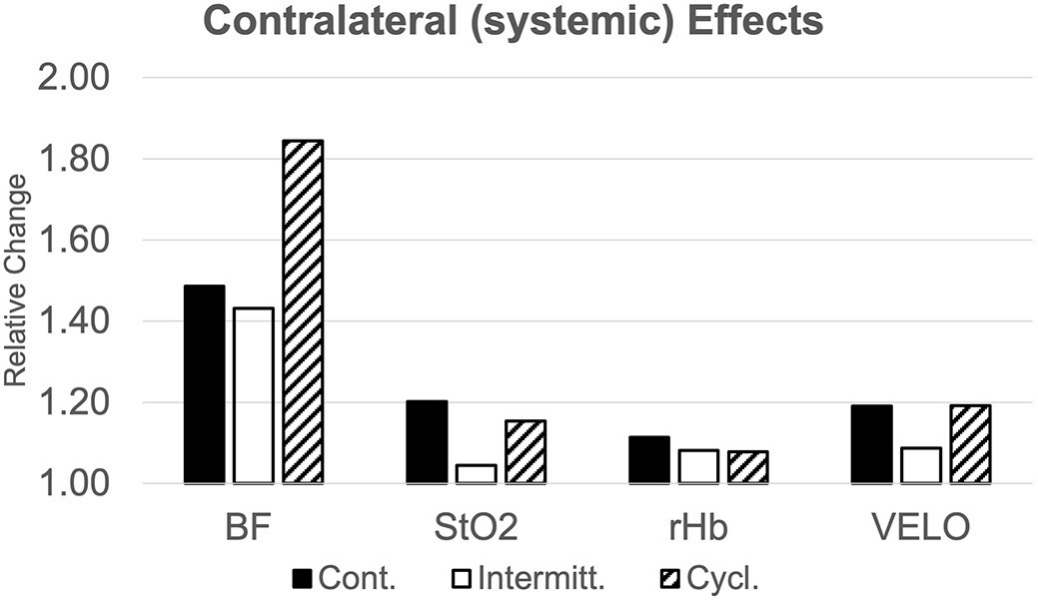
Remote effect of NPWT. Relative changes of microcirculatory parameters compared to baseline values on the contralateral thigh 60-minutes after activation of the NPWT-device (BF, Bloodflow; StO_2_, Tissue Oxygen Saturation; rHb, relative Hemoglobin Content; VELO, Red Blood Cell Velocity).

Cutaneous microcirculation of the contralateral thigh was also affected by NPWT treatment. We observed a similar virtually linear increase in BF 90 min after BL in all groups. This almost linear increase was most pronounced in group 3. StO_2_ values in group 1 increased almost twice than those of groups 2 and 3. In addition, BF changes reached statistical significance compared to BL values, while differences between groups did not. Changes in rHb showed an almost significant difference between groups (*p* = 0.005) with an increase of +13.0% (CI: 1.088–1.172) in group 1 compared to +8.6% (1.054–1.118) for group 2 and +6.9% (1.044–1.094) for group 3. VELO was significantly altered in all three groups with groups 1 and 3 showing an increase nearly twice as high as the increase of group 2. See Figure 6 for visualization of remote effects at the end of active-suction period 60 min after BL. Comprehensive data of contralateral perfusion measurements is available online as a supplementary file.

## Discussion

Within this preclinical study on acute changes of cutaneous microcirculation under an applied NPWT dressing, we observed a significant increase of local perfusion dynamics with consecutive improvement of tissue oxygen saturation. Interestingly, all three compared modes of application, continuous, intermittent, and cyclic, resulted in locally enhanced microcirculation of a greater or lesser extent. In the comparison of different application modes, we observed superior effects on local and remote cutaneous perfusion in the cyclic group.

The real-time assessment of changes in cutaneous perfusion utilizing combined laser-doppler and white-light spectroscopy (“O2C-decvice”) represents an established method in the field of microcirculatory research within soft tissues (11). Due to the combination of both techniques and different parameters, a comprehensible insight of acute changes in the capillary compartment can be achieved. In addition, continuous data recording allows for the evaluation of mid and long-term effects of specific stimuli. The three tested modes of application represent the typical therapy options of commonly available NPWT devices in the market. On the other hand, the continuous mode represented the most common setting in clinical wound care according to a published meta-analysis of Suissa et al. in 2011, in which discontinuous applications were rarely reported (12). To the best of our knowledge, there is no other recent systematic evaluations of currently used treatment algorithms have been published. Notably, continuous treatment represents the generally accepted standard of care despite already available early evidence of superior capabilities of an intermittent NPWT treatment with respect to formation of granulation tissue or angiogenesis (3). Most likely, this is attributable to the fact that intermittent activation of “negative-pressure,” which causes repeated spikes in surface pressure to the wound, is believed to be unpleasing. Although this assumption might reflect the clinical experience of most physicians, published data on this issue is rare. Lately, the introduction of the “cyclic-mode” appears as a promising compromise combining both the satisfaction of patients and superior wound treatment (13).

Although reported pain levels were generally low, we observed a strong correlation between resulting surface pressure and pain sensation in our study. Of note, the course of induced discomfort mirrors the mode of applied suction, and thus could be accurately sensed by participants. The changes in surface pressure resulting from different amounts of suction within groups reached statistical significance, indicating a relevant difference of applied forces to the underlying tissue. Although we used a different device in our current study, examined values were comparable to previous investigations in our department (8). An almost linear increase in surface pressure was found in relation to applied suction, which is also in line with results published earlier (10, 14).

In human cutaneous microcirculation, resting capillary pressure was reported in a range from 10.5 to 22.5 mmHg or even 41.0 mmHg (15, 16). Thus, applied surface pressure of ~30.0 mmHg via a NPWT dressing could potentially result in an occlusion of cutaneous capillaries. Given the finding that capillary pressure also increases in response to a higher venous pressure, at least a sub-total occlusion of the dermal microvasculature due to the intervention can be assumed (17). On the other hand, this response might explain our findings of a continuous increase in local perfusion in the continuous group. Overall, the mechanisms of cutaneous vascular response to certain stimuli are complex, especially concerning vasodilation and improvement of local flow (18). Repeated capillary (sub-total) occlusion represents a strong stimulus for the affected tissue. Both post-occlusive reactive hyperemia (PORHA) and increased mechano-humoral transduction to the vascular bed result in alterations of intravascular shear stress and could be accountable for superior effects in the intermittent and, particularly, in the cyclic group (19, 20).

We also assessed changes of cutaneous microcirculation on the contralateral thigh and found stronger effects in the cyclic group. From previous studies on Remote Ischemic Conditioning (RIC), we know how alterations in the applied stimulus can influence the triggered improvement of cutaneous perfusion (21, 22). Duration of applied pressure, number of repeated cycles, and body site are important variables to optimize the conditioning effect on the improvement of remote microcirculation. That being said, our results implicitly back up the assumption for great relevance of the elicited stimulus by indicating a more condensed and, therefore, more powerful triggering in the cyclic group. Morykwas and Argenta already demonstrated superior effects of an intermittent application of negative pressure with regards to the formation of granulation tissue in their initial publications (23). Further studies have attributed those beneficial effects to higher levels of growth factors and activation of mechanosensitive gene-expression (20).

Our results are limited due to the fact that we only included young healthy individuals and that measurements were carried out on intact skin. With regards to the clinical setting and the treatment of a variety of wounds, microcirculatory response of an actual wound bed is supposed to be influenced by several factors like age, preexisting comorbidities, nicotine consumption, drugs, or conducted surgeries, among others. Moreover, wound etiology and infectious status are typical variables of pivotal importance for perfusion characteristics and wound healing in general. Altogether, results obtained in this preclinical study might vary from observations under clinical conditions. As discussed by several authors, we agree that an ideal application of a NPWT dressing must respect the individual circumstances of each patient and treated wounds with respect to comorbidities, location of the wound, and tissue composition (24). However, with our study, we expand the understanding of microcirculatory changes in soft tissues subjected to NPWT dressing. Moreover, our results could be of high interest regarding application of closed incision Negative Pressure Therapy devices (ciNPT). A recent systematic Cochrane library review found large variance in the amount of applied suction and duration using ciNPT. Moreover, an international multidisciplinary consensus statement is lacking a recommendation regarding the mode of application or settings for negative pressure (25, 26).

## Conclusion

Cyclic application of “negative pressure” results in a superior local enhancement of cutaneous microcirculation with regards to blood flow and consecutive tissue oxygenation. Beyond that, repeated alterations between different levels of “negative pressure” due to cyclic application represent a greater stimulus for remote conditioning effects, indicating a superior local interaction with the underlying tissue.

Further research is warranted to investigate the correlation between local perfusion enhancements and granulation tissue formation due to continuous vs. cyclic NPWT in humans.

## Data Availability Statement

The original contributions presented in the study are included in the article/supplementary material, further inquiries can be directed to the corresponding author/s.

## Ethics Statement

The studies involving human participants were reviewed and approved by Ethik-Kommission der Medizinischen Fakultät der Ruhr-Universität Bochum. The patients/participants provided their written informed consent to participate in this study.

## Author Contributions

AS and BB contributed to the conceptualization of the study. AS and MD contributed to the methodology. AS conducted the investigation while BB and ML conducted the formal analysis. ML and BB contributed with resources. AS contributed to the writing of the original draft and MD, CW, JW, and MvG reviewed and edited the paper. AS contributed to the visualization of the study and assisted with data curation. CW and BB supervised the whole process and AS was in charge of project administration. Lastly, AS and ML contributed to the funding acquisition. All authors have read and agreed to the published version of the manuscript. All authors contributed to the article and approved the submitted version.

## Funding

AS received a grant from the Junior Clinician Scientist program by the FoRUM-program of the medical faculty of the Ruhr-University Bochum (K128–19).

## Conflict of Interest

The authors declare that the research was conducted in the absence of any commercial or financial relationships that could be construed as a potential conflict of interest.

## Publisher's Note

All claims expressed in this article are solely those of the authors and do not necessarily represent those of their affiliated organizations, or those of the publisher, the editors and the reviewers. Any product that may be evaluated in this article, or claim that may be made by its manufacturer, is not guaranteed or endorsed by the publisher.
